# What is the lifetime cost of alcohol consumption? an estimation of economic burden in Thailand

**DOI:** 10.1371/journal.pone.0322944

**Published:** 2025-05-16

**Authors:** Chaisiri Luangsinsiri, Montarat Thavorncharoensap, Usa Chaikledkaew, Oraluck Pattanaprateep, Naiyana Praditsitthikorn, Bundit Sornpaisarn, Jürgen Rehm

**Affiliations:** 1 Doctor of Philosophy Program in Social, Economic, and Administrative Pharmacy, Department of Pharmacy, Faculty of Pharmacy, Mahidol University, Bangkok, Thailand; 2 Institute for Mental Health Policy Research, Centre for Addiction and Mental Health, Toronto, Canada; 3 Mahidol University Health Technology Assessment (MUHTA) International Graduate Program, Mahidol University, Bangkok, Thailand; 4 Social and Administrative Pharmacy Excellence Research (SAPER) unit, Department of Pharmacy, Faculty of Pharmacy, Mahidol University, Bangkok, Thailand; 5 Department of Clinical Epidemiology and Biostatistics, Faculty of Medicine Ramathibodi Hospital, Mahidol University, Thailand; 6 Department of Disease Control, Ministry of Public Health, Nonthaburi, Thailand; 7 Dalla Lana School of Public Health, University of Toronto, Toronto, Canada; Kandahar University, Faculty of Medicine, AFGHANISTAN

## Abstract

This study aimed to estimate the lifetime cost of alcohol consumption per individual drinker in Thailand to support policy formulation. Using an incidence-based cost-of-illness (COI) approach, a hybrid model combining a decision tree and a Markov model, incorporating six major alcohol-related diseases and conditions (i.e., hypertension, hemorrhagic stroke, liver cirrhosis, liver cancer, alcohol use disorders, and road injuries), was employed to analyze both direct costs (i.e., direct medical, direct nonmedical, property damage) and indirect costs (i.e., absenteeism, premature mortality). All costs were reported in Thai baht 2022 (35.06 THB = 1US$). From a societal perspective, the lifetime costs for individual male and female drinker were estimated at 721,344 THB (95% CI: 687,910–754,779) and 263,812 THB (95% CI: 249,250–278,374), respectively. Quitting earlier reduced costs significantly, with average quitting ages resulting in the cost of 568,932 THB for males and 115,167 THB for females. On average, each Thai drinker incurs a cost of 498,196 THB. These findings highlight the substantial economic burden of alcohol consumption in Thailand, underscoring the critical need for effective interventions and policies, along with more rigorous enforcement of current regulations aimed at encouraging early cessation and preventing the initiation of drinking, such as through advertising bans, sales restrictions, improving access to counseling and treatment.

## Introduction

Alcohol consumption is one of the major risk factors for chronic diseases and injuries, leading to substantial burden of disease, globally [1,2]. In 2019, the last year before the start of the COVID-19 pandemic, approximately 2.5 billion people worldwide consumed alcohol, and 400 million individuals suffered from alcohol use disorders (AUDs) [1]. In the same year, it was estimated that alcohol use was responsible for 4.7% of all deaths and 4.6% of disability-adjusted life years (DALYs) [1]. Given its numerous negative effects on health, alcohol consumption is inevitably responsible for substantial economic burden [3–5].

Estimating the economic burden of alcohol consumption is crucial for aiding in policy decision-making and prioritization, as well as for helping to raise societal awareness regarding the harmful impact of alcohol use. To estimate the economic burden of alcohol, cost-of-illness (COI) analyses are employed [6–8]. Results of the COI studies are expressed in monetary values representing the total economic burden of alcohol consumption on society. It should be noted that there are two types of approaches when undertaking a COI study [9]: prevalence-based and incidence-based. The prevalence-based approach focuses on estimating the costs attributed to alcohol consumption occurring within a given year. In contrast, the incidence-based approach provides estimates on the lifetime costs or the costs incurred by each individual alcohol drinker over his/her lifetime. While the findings from prevalence-based studies can be used to raise awareness of the economic burden of alcohol consumption on society and to assist in policy planning and resource allocation, incidence-based studies offer useful information for estimating the cost-effectiveness of policies/interventions aimed at reducing the incidence/prevalence of alcohol consumption.

To date, several efforts have been made globally to estimate the economic burden of alcohol using a prevalence-based approach [4,5]. According to the most recent study, economic costs of alcohol consumption, estimated using a prevalence-based approach, was equivalent to 2.6% of GDP [5]. However, incidence-based studies on the costs of alcohol consumption are much rarer, with only one published study identified [10]. In that study [10], the target was a UK male, aged 40 years old. The Markov model consisted of five health states (i.e., harmful drinking, hazardous drinking, ex-harmful drinking, ex-hazardous drinking, and death). The model outcomes included years of life, quality-adjusted life year (QALY), and lifetime costs associated with the intervention under evaluation [10].

Alcohol consumption remains a significant risk factor in Thailand accounting for 9.8% of DALY losses in 2019 [11]. According to the recent national survey conducted in 2021, an estimated 28.0% of the Thai population were identified as being current drinkers (46.4% in males and 10.8% in females) [12]. The prevalence of current alcohol drinkers among Thai adolescents was also found to have increased from 14.8% in 2008 to 22.2% in 2015 [13]. Furthermore, it was reported that the prevalence of alcohol consumption and heavy episodic drinking among Thai adolescents was highest in Southeast Asia^.^[1]. Previous prevalence-based COI studies on the economic costs of alcohol consumption in Thailand [14–16] demonstrated that the economic burden of alcohol use in the country ranged from 0.56% to 1.99% of the country’s GDP. Only one incidence-based COI study, which considered only the cost of premature mortality, was conducted in Thailand, 2011 [17]. The results of the study found that the lifetime cost of harmful use of alcohol consumption was 360,000 baht, and 240,000 baht (value in year 2010) for males and females, respectively.

To support policy decisions aimed at reducing alcohol prevalence/incidence in the country, there is a need for more comprehensive research to estimate the economic costs of alcohol consumption in Thailand. Unfortunately, evidence regarding the lifetime cost imposed by each drinker globally, including in Asia, is scarce. As mentioned earlier, only 2 studies have been identified [10,17]. However, it is important to point out that the drinking patterns (i.e., hazardous drinking and harmful drinking) examined in the previous UK study may not be applicable to typical drinking behavior in Thailand [10]. Additionally, the previous study was conducted in Western country with different cultural and economic context [10]. It is also crucial to highlight that the previous study did not report lifetime costs for individual drinker, as it only provided the cost-effectiveness evidence of the intervention designed to alter drinking behavior, based on findings from the COI study [10]. Furthermore, another incidence-based COI study conducted in Thailand considered only the indirect cost associated with premature mortality due to drinking [17]. Thus, this study aimed to estimate the lifetime cost of alcohol consumption for an individual in Thailand, considering both direct and indirect costs. Specifically, we aimed to estimate the economic costs incurred by the following types of drinkers in Thailand: 1) the individual drinker who drinks throughout his/her life (i.e., lifetime drinker); 2) the individual drinker who quits at a specific age (i.e., 35. 45, 55, and 65 years old); 3) the individual drinker who quits at the average quitting age in Thailand (i.e., 55 and 49 years old for males and females, respectively) [12]; and 4) the average individual drinker. Additionally, it should be noted that the drinking pattern of the population in the model was tailored to reflect a Thai-specific drinking pattern. The study’s findings could play a key role in providing evidences to support policies focused on preventing the initiation of drinking and promoting earlier cessation.

## Methods

### Study design

The detailed methodology of this study can be found in a previous publication [18]. In summary, this study is an incidence-based COI analysis.

### Model structure

A hybrid model, using a decision tree and Markov model, was employed. A Markov model was developed to predict the lifetime costs of alcohol consumption as well as life expectancy of a Thai male drinker who started drinking at the age of 20 years old consuming an average of 30.1 gram of alcohol/day and a female drinker who started drinking at the age of 23 years old, consuming an average of 8.0 gram/day, which is representative of the current drinking behavior in Thailand [12,19]. In addition, the costs to society resulting from the actions of an individual drinker who quits at a specific age (i.e., 35, 45, 55, and 65 years old) and the average age of quitting for Thai drinkers (i.e., 55, and 49 years old for males and females, respectively) were also estimated in the scenario analyses.

Given the limitations of epidemiological parameters and to prevent the model from becoming overly complex, six major alcohol-related diseases/conditions (i.e., hypertension, hemorrhagic stroke, liver cirrhosis, liver cancer, AUDs, and road injuries) were considered in the Markov model [18]. According to the model structure, healthy individuals can develop hypertension, liver cirrhosis, liver cancer, AUDs, and road injuries with the annual probability estimated from the general Thai population. Such probabilities varied depending on the drinking status (i.e., abstainer, current drinker, and former drinker). Given that alcohol-related diseases are the result of accumulated years of alcohol consumption, the time lags from alcohol drinking to the development of the following diseases were set in the analysis as follows: 20 years for liver cirrhosis/liver cancer, 10 years for hypertension/hemorrhagic stroke [20], and 8 years for AUDs [21]. However, the time lag for getting a road injury as a result of alcohol consumption was set at zero as alcohol intoxication instantly affects driving ability [2]. Assumptions along with the calculation of annual transitional probability can be found in the previous publication [18].

### Costs

Both direct and indirect costs were considered in our analysis. Direct costs consisted of direct medical costs, direct non-medical costs, and direct costs related to property damage from road traffic accidents. Indirect costs comprised indirect costs of hospital admission-related absenteeism, and indirect costs related to premature mortality. The direct medical costs estimated from the analysis represent the costs from a healthcare perspective. The sum of all costs represents the costs from a societal perspective. All costs were reported in Thai baht (THB), 2022 (35.06 THB = 1 US$) [22].

### Data analysis

**Fig 1:**
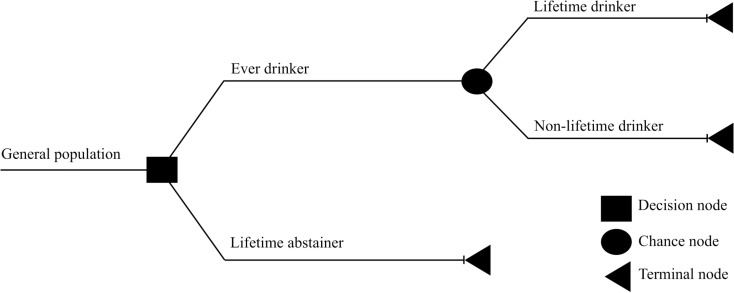
The decision-tree model.

Lifetime costs of alcohol consumption were estimated as the excess costs incurred for a lifetime drinker, compared to a lifetime abstainer. Costs saved from quitting drinking were estimated to be the difference between costs to society of a lifetime drinker versus costs incurred by a drinker who quits at a specific age. To predict the cost incurred by the society for each individual new drinker in Thailand, the findings from the Markov model were then linked to a decision tree (Fig 1). As shown in Fig 1, the general Thai population can be divided into two distinct groups: lifetime abstainers and ever drinkers. Ever drinkers can be further divided into subgroups of lifetime drinkers and non-lifetime drinkers. In our analysis, a lifetime drinker was defined as a drinker who continued to drink at the age of 70 years and above. A non-lifetime drinker, in contrast, was assumed to be a drinker who quits drinking at age 55 and 49 years old, for a male and a female, respectively. These ages were determined based on the average age of former drinkers in Thailand [12]. The cost of the individual drinker was then estimated to be the difference between the cost of a lifetime abstainer and an ever drinker.

Sensitivity analyses were conducted to determine whether the results are robust to parameter uncertainty. Full details on the sensitivity analysis can be found in the previous literature [18]. Briefly, a one-way sensitivity analysis was performed by varying input parameters such as amount of daily alcohol intake, relative risks, discount rates, and income growth rates. A probabilistic sensitivity analysis (PSA) using Monte Carlo simulations with 1,000 iterations was also conducted. Beta distribution was implemented for the transitional probabilities, while gamma distribution was used for the cost parameters. Model validation was performed by comparing the age-specific survival data and life expectancy estimated from the hybrid model with those of the general Thai population. All transitional probabilities [2,12,20,23–44] were reported in Table 1 and the other parameters used in the analysis along with its sources were summarized in S1 Table in S1 File. Ethical approval for this study was granted by the Institutional Review Board of Mahidol University (COE.No.MU-DT/PY-IRB 2021/010.0605).

**Table 1:** Details on the transitional probabilities.

Parameter	Value^*^		Reference
	Male	Female	
Annual transitional probabilities of developing alcohol-related diseases			
1. The probabilities for lifetime abstainers			
Healthy to hypertension	0.00534-0.02683	0.00273-0.01684	[23–25]
Healthy to hemorrhagic stroke	0.00002-0.00074	0.00002-0.00057	[12,23,26–28]
Healthy to liver cirrhosis	0.00001-0.00160	0.00001-0.00134	[12,23,29]
Healthy to liver cancer	1.07x10^-6^-8.47x10^-5^	7.52x10^-7^-6.30x10^-5^	[12,23,30,31]
Healthy to AUDs	0.00000	0.00000	[2]
Healthy to non-fatal injury	0.00026-0.00063	0.00037-0.00148	[12,23,32]
Hypertension to hemorrhagic stroke	0.00092-0.00137	0.00124-0.00141	[12,26,27,34]
Hypertension to liver cirrhosis	0.00001-0.00160	0.00001-0.00134	Assumption
Hypertension to liver cancer	1.07x10^-6^-8.47x10^-5^	7.52x10^-7^-6.30x10^-5^	Assumption
Liver cirrhosis to liver cancer	0.01980-0.02030	0.02837-0.02856	[12,33,35]
Non-fatal injury to healthy	1.00000	1.00000	Assumption
2. The probabilities for lifetime drinkers			
Healthy to hypertension	0.00705-0.03543	0.00286-0.01765	[20,23–25]
Healthy to hemorrhagic stroke	0.00003-0.00101	0.00003-0.00078	[12,20,23,26–28]
Healthy to liver cirrhosis	0.00002-0.00397	0.00002-0.00200	[12,20,23,29]
Healthy to liver cancer	1.69x10^-6^-1.34x10^-4^	1.19x10^-6^-9.95x10^-5^	[12,20,23,30,31]
Healthy to AUDs	0.00059-0.05774	0.00084-0.01537	[12,21,23]
Healthy to non-fatal road injury	0.00759-0.02068	0.00046-0.01037	[12,23,32]
Hypertension to hemorrhagic stroke	0.00190-0.00282	0.00255-0.00291	[12,20,26,34]
Hypertension to liver cirrhosis	0.00002-0.00397	0.00002-0.00200	Assumption
Hypertension to liver cancer	1.69x10^-6^-1.34x10^-4^	1.19x10^-6^-9.95x10^-5^	Assumption
Liver cirrhosis to liver cancer	0.02079-0.02131	0.02979-0.02999	[12,20,33,35]
AUDs to hypertension	0.01394-0.07441	0.00643-0.04045	[20,23,36]
AUDs to hemorrhagic stroke	0.00003-0.00101	0.00003-0.00078	Assumption
AUDs to liver cirrhosis	0.00008-0.02741	0.00019-0.02598	[20,23,37]
AUDs to liver cancer	1.69x10^-6^-1.34x10^-4^	1.19x10^-6^-9.95x10^-5^	Assumption
AUDs to non-fatal road injury	0.00759-0.02068	0.00046-0.01037	Assumption
AUDs to healthy	0.06574	0.06574	[38]
Non-fatal injury to healthy	1.00000	1.00000	Assumption
Non-fatal injury with AUDs to healthy	1.00000	1.00000	Assumption
3. The probabilities for former drinkers			
Healthy to hypertension	0.00534-0.02683	0.00273-0.01684	[20,23–25]
Healthy to hemorrhagic stroke	0.00002-0.00074	0.00002-0.00057	[12,20,23,26–28]
Healthy to liver cirrhosis	0.00001-0.00160	0.00001-0.00134	[12,20,23,29]
Healthy to liver cancer	1.07x10^-6^-8.47x10^-5^	7.52x10^-7^-6.30x10^-5^	[12,20,23,30,31]
Healthy to AUDs	0.00000	0.00000	[2]
Healthy to non-fatal road injury	0.00026-0.00063	0.00037-0.00148	[12,23,32]
Hypertension to hemorrhagic stroke	0.00092-0.00137	0.00124-0.00141	[12,20,26,34]
Hypertension to liver cirrhosis	0.00001-0.00160	0.00001-0.00134	Assumption
Hypertension to liver cancer	1.07x10^-6^-8.47x10^-5^	7.52x10^-7^-6.30x10^-5^	Assumption
Liver cirrhosis to liver cancer	0.01980-0.02030	0.02837-0.02856	[12,20,33,35]
AUDs to healthy	1.00000	1.00000	Assumption
Non-fatal injury to healthy	1.00000	1.00000	Assumption
Non-fatal injury with AUDs to healthy	1.00000	1.00000	Assumption
Annual transitional probabilities of death			
Hypertension to death	0.01474-0.13687	0.01174-0.10319	[39,40]
Fatal hemorrhagic stroke	0.13742-1.00000	0.10938-0.98774	[27]
Post-stroke to death	0.02010-0.19337	0.01600-0.14446	[27,40]
Liver cirrhosis to death	0.05704-0.50451	0.04533-0.36546	[40,41]
Liver cancer to death	0.12912-1.00000	0.10277-0.92809	[40,42]
AUDs to death	0.00246-0.02365	0.00196-0.01767	[43]
Fatal injury among AUDs	0.00067-0.00645	0.00053-0.00482	[43]
Fatal injury among lifetime abstainers	1.32x10^-6^-2.23x10^-4^	3.99x10^-6^-2.88x10^-4^	[12,23,40,44]
Fatal injury among lifetime drinkers	0.00028-0.02596	0.00017-0.00276	[12,23,40,44]
Fatal injury among former drinkers	1.32x10^-6^-2.23x10^-4^	3.99x10^-6^-2.88x10^-4^	[12,23,40,44]
Death from the other causes	0.00040-0.12421	0.00010-0.09149	[12,23,40]

*Value varied by age.

## Results

Table 2 presents the predicted life expectancies and total lifetime cost of alcohol consumption of an individual by gender and drinking status. When considering six alcohol-related diseases, the analysis revealed that the lifetime male drinker and female drinker had 2.66 years, and 1.21 years shorter life expectancy compared to lifetime abstainer, respectively. From a healthcare payer perspective, the analysis revealed that an individual lifetime drinker incurred excess direct medical costs of 56,035 (95% CI: 53,747–58,324) baht for females and 205,766 (95% CI:195,750–215,783) baht for males, compared to a lifetime abstainer. From a societal perspective, each female drinker who drank alcohol throughout her lifetime brought about an excess cost of 263,812 (95% CI: 249,250–278,374) baht. Excess cost incurred by each male lifetime drinker under societal perspective was estimated to be 721,344 (95%CI: 687,910–754,779) baht.

**Table 2:** Predicted life expectancy and the economic costs of alcohol consumption in Thailand by type of drinkers and perspective in baht (2022).

Scenario	Predicted life expectancy (years)	∆ in life expectancy^*^ (years)	Healthcare perspective (baht)				Societal perspective (baht)					
			Total cost	∆ in total cost^*^	95% CI		Total cost	∆ in total cost^*^		95% CI		
					Lower	Upper				Lower		Upper
**Male**												
1. LA	76.16		168,727				1,556,840					
2. LD	73.50	-2.66	374,494	205,766	195,750	215,783	2,278,184	721,344	687,910		754,779	
3. Q35	75.39	1.89	193,397	-181,096	-190,036	-172,157	1,748,896	-529,288	-551,558		-507,018	
4. Q45	74.61	1.11	244,042	-130,452	-136,953	-123,950	1,959,087	-319,097	-331,723		-306,471	
5. Q55	73.98	0.48	295,226	-79,267	-83,453	-75,081	2,125,772	-152,412	-158,274		-146,550	
6. Q65	73.72	0.22	332,386	-42,107	-44,416	-39,799	2,210,903	-67,281	-70,106		-64,456	
**Female**												
1. LA	81.29		142,357				1,434,560					
2. LD	80.08	-1.21	198,393	56,035	53,747	58,324	1,698,372	263,812	249,250		278,374	
3. Q35	81.15	1.06	144,590	-53,803	-56,013	-51,593	1,465,507	-232,865	-245,673		-220,058	
4. Q45	80.96	0.88	154,389	-44,004	-45,825	-42,183	1,515,837	-182,535	-192,641		-172,429	
5. Q49	80.90	0.81	161,081	-37,312	-38,859	-35,765	1,549,727	-148,645	-156,711		-140,579	
6. Q55	80.83	0.74	170,049	-28,344	-29,521	-27,167	1,588,589	-109,783	-115,620		-103,947	
7. Q65	80.68	0.59	183,147	-15,245	-15,917	-14,574	1,641,399	-56,973	-60,475		-53,471	

LD Lifetime abstainer; LA Lifetime drinker; Q35 Quit at 35; Q45 Quit at 45; Q49 Quit at 49; Q55 Quit at 55; Q65 Quit at 65.

*for LD, the change was compared to LA; for Q35, Q45, Q49, Q55, and Q65, the change was compared to LD.

As shown in Table 2, life-years and costs saved by quitting, compared to lifetime drinking, were higher when an individual quit sooner rather than later in life. Our estimates revealed that the costs saved by the individual drinker who quits at the age of 35 ranged from 232,865 for females and 529,288 baht for males, while those for an individual drinker who quit at the age of 65 ranged from 56,973and 67,281 baht, for females and males, respectively. The excess costs incurred by an individual Thai drinker who quit at the average quitting age in Thailand (55 years old for a male and 49 years old for a female) compared to a lifetime abstainer was estimated at 568,932 baht for male and 115,167 baht for female, respectively.

Table 3 presents the costs of alcohol drinking by drinking status and cost components. For each drinking status, it was consistently found that the cost of premature mortality represented the largest proportion of the total cost, followed by direct medical costs, and indirect costs of absenteeism, respectively. The costs of premature mortality accounted for the largest component of excess costs among lifetime male drinkers (58.02% of the total excess costs) followed by direct medical costs (28.53% of the total excess costs), and cost of absenteeism (10.89% of the total excess cost), respectively. The largest component of the costs saved from quitting drinking for male drinkers depends on the age he quits drinking at. If male drinkers quit at a young age, the majority of the cost savings are due to a reduction in premature mortality cost, whereas quitting at an older age leads to more significant savings in healthcare costs. For the male drinker, savings from the reduction in healthcare costs ranged from 34.22% of the total savings (quit at the age of 35) and 62.58% of the savings (quit at the age of 65). Conversely, the costs saved due to the reduction in premature mortality varied from 49.75% of the total saved costs (quit at the age of 35) baht to 9.34% of the total saved costs (quit at the age of 65). Nevertheless, for female drinkers, the costs saved from premature mortality represented the major contributor to the total excess costs, followed by the direct medical costs, regardless of the age when they quit.

**Table 3:** Economic costs of alcohol consumption for each type of drinkers by cost component in baht (2022).

Scenario	Direct cost						Indirect cost				Total cost	∆ in total cost^*^
	Direct medical cost (% of total cost)	∆ in direct medical cost (% of ∆ in total cost)^*^	Direct non-medical cost (% of total cost)	∆ in direct non-medical cost (% of ∆ in total cost)^*^	Cost of property damage (% of total cost)	∆ in cost of property damage (% of ∆ in total cost)^*^	Cost of absenteeism (% of total cost)	∆ in cost of absenteeism (% of ∆ in total cost)^*^	Cost of premature mortality (% of total cost)	∆ in cost of premature mortality (% of ∆ in total cost)^*^		
**Male**												
1. LA	168,727 (10.84)		13,979 (0.90)		13 (0.00)		66,945 (4.30)		1,307,175 (83.96)		1,556,840	
2. LD	374,494 (16.44)	205,766 (28.53)	32,224 (1.41)	18,245 (2.53)	302 (0.01)	290 (0.04)	145,464 (6.39)	78,519 (10.89)	1,725,700 (75.75)	418,524 (58.02)	2,278,184	721,344
3. Q35	193,397 (11.06)	-181,096 (34.22)	16,170 (0.92)	-16,053 (3.03)	202 (0.01)	-100 (0.02)	76,774 (4.39)	-68,690 (12.98)	1,462,353 (83.62)	-263,347 (49.75)	1,748,896	-529,288
4. Q45	244,042 (12.46)	-130,452 (40.88)	20,955 (1.07)	-11,269 (3.53)	256 (0.01)	-46 (0.01)	97,255 (4.96)	-48,210 (15.11)	1,596,579 (81.50)	-129,121 (40.46)	1,959,087	-319,097
5. Q55	295,226 (13.89)	-79,267 (52.01)	25,407 (1.20)	-6,817 (4.47)	277 (0.01)	-26 (0.02)	116,541 (5.48)	-28,924 (18.98)	1,688,321 (79.42)	-37,379 (24.52)	2,125,772	-152,412
6. Q65	332,386 (15.03)	-42,107 (62.58)	28,608 (1.29)	-3,615 (5.37)	288 (0.01)	-14 (0.02)	130,206 (5.89)	-15,259 (22.68)	1,719,415 (77.77)	-6,285 (9.34)	2,210,903	-67,281
**Female**												
1. LA	142,357 (9.92)		12,376 (0.86)		34 (0.00)		57,868 (4.03)		1,221,924 (85.18)		1,434,560	
2. LD	198,393 (11.68)	56,035 (21.24)	15,809 (0.93)	3,433 (1.30)	209 (0.01)	175 (0.07)	73,551 (4.33)	15,683 (5.94)	1,410,410 (83.04)	188,486 (71.45)	1,698,372	263,812
3. Q35	144,590 (9.87)	-53,803 (23.10)	12,463 (0.85)	-3,346 (1.44)	103 (0.01)	-107 (0.05)	58,317 (3.98)	-15,234 (6.54)	1,250,034 (85.18)	-160,376 (68.87)	1,465,507	-232,865
4. Q45	154,389 (10.19)	-44,004 (24.11)	13,139 (0.87)	-2,670 (1.46)	140 (0.01)	-69 (0.04)	61,627 (4.07)	-11,924 (6.53)	1,286,541 (85.30)	-123,869 (67.86)	1,515,837	-182,535
5. Q49	161,081 (10.39)	-37,312 (25.10)	13,559 (0.87)	-2,250 (1.51)	151 (0.01)	-58 (0.04)	63,469 (4.10)	-10,082 (6.78)	1,311,467 (84.63)	-98,942 (66.56)	1,549,727	-148,645
6. Q55	170,049 (10.70)	-28,344 (25.82)	14,115 (0.89)	-1,694 (1.54)	166 (0.01)	-44 (0.04)	65,912 (4.15)	-7,639 (6.96)	1,338,347 (84.25)	-72,063 (65.64)	1,588,589	-109,783
7. Q65	183,147 (11.16)	-15,245 (26.76)	14,894 (0.91)	-916 (1.61)	186 (0.01)	-24 (0.04)	69,396 (4.23)	-4,155 (7.29)	1,373,776 (83.70)	-36,633 (64.30)	1,641,399	-56,973

LD Lifetime abstainer; LA Lifetime drinker; Q35 Quit at 35; Q45 Quit at 45; Q49 Quit at 49; Q55 Quit at 55; Q65 Quit at 65.

*for LD, the change was compared to LA; for Q35, Q45, Q49, Q55, and Q65, the change was compared to LD.

A breakdown of costs attributed to each alcohol-related disease for lifetime drinkers and lifetime abstainers is provided in S2 Table in S1 File. Regardless of gender, the most significant contributor to the total costs in both lifetime abstainers and drinkers was hypertension. However, when looking at the excess costs, AUDs accounted for the largest share of the total excess cost of alcohol consumption (40.86% of the total excess cost) for male lifetime drinkers, followed by hypertension (33.55% of the total excess cost), and road injuries (14.29% of the total excess cost). The top leading cause of the total excess cost for lifetime female drinkers, however, was road injury (35.11% of the total excess cost), followed by AUDs (21.04% of the total excess cost), and hemorrhagic stroke (16.57% of the total excess cost). For non-lifetime drinkers, hypertension emerged as the primary contributor to the total excess cost of alcohol consumption for males, constituting 39.49% of the total excess costs, followed by AUDs (34.84% of the total excess cost) and road injuries (17.03% of the total excess costs). Road injury, however, took the lead as the major cause of the total excess cost among female non-lifetime drinkers, making up 42.75% of the total excess cost, followed by AUDs (21.02% of the total excess cost) and hypertension (15.05% of the total excess cost).

As shown in Table 4, estimated costs incurred for each individual male drinker ranged from 145,988 baht under healthcare payer perspective to 606,404 baht from the societal perspective. Costs incurred for each individual female drinker from the healthcare-payer and societal perspectives, respectively, were estimated to be 26,625 baht and 146,646 baht. Given that 76.46% of Thai drinkers were male, the cost for each individual Thai drinker on average was estimated to be 117,895 baht from the healthcare payer perspective and 498,196 baht from the societal perspective.

**Table 4:** Estimation of cost of individual Thai drinker.

Gender	Healthcare perspective (baht)	Societal perspective (baht)
Male	145,988	606,404
Female	26,625	146,646
**Average**	**117,895**	**498,196**

The results of the one-way sensitivity analysis can be found in S1-S10 Figs in S1 File. Our analysis revealed that the discount rate and the choice of the income growth rate were the two main parameters that had the largest impact on the lifetime costs. For model validation, the survival curve for the general Thai population and that of an individual by their drinking status and gender is reported in S11-S12 Figs in S1 File.

## Discussion

In our analysis, the lifetime costs of each particular type of alcohol drinker in Thailand were estimated from both healthcare and societal perspectives. Our study revealed that, on average, the cost for each individual Thai drinker was estimated to be 117,895 baht from the healthcare payer perspective and 498,196 baht from the societal perspective. Among lifetime drinkers, indirect costs, particularly cost of premature mortality, accounted for the majority of the economic burden. The significant lifetime costs estimated emphasize the urgent need for interventions to prevent the initiation of alcohol consumption, particularly through the restriction of alcohol marketing, which is considered the most effective policy for promoting alcohol abstention [45]. Furthermore, considering the high proportion of premature deaths, interventions focused on reducing alcohol-related fatalities, particularly among younger populations, are essential.

While a recent prevalence-based COI study in Thailand from 2021 [46] reported that the economic burden of alcohol accounted for 1.02% of GDP, our lifetime cost estimated from the incidence-based approach cannot be directly compared to GDP. Unlike the prevalence-based approach, which calculates the annual costs related to alcohol consumption in a specific setting, the incidence-based approach assesses the lifetime cost per individual drinker. Findings from the prevalence-based approach are often compared to national economic indicators, such as GDP, to highlight the scale of the burden and facilitate comparisons across different contexts. However, the results from the incidence-based approach reflected costs accumulated over a lifetime, not just within a single year. Therefore, comparing these results to GDP, which represents the total value of all goods and services produced within a country in a given year, may not be appropriate. This limitation should be acknowledged as a challenge when comparing incidence-based results across countries.

Compared to the previous study in Thailand [17] that examined the lifetime costs, using an incidence-based COI approach, the results of this study produced higher estimates. While the previous study reported the indirect cost (2022 value) of lifetime light drinker as ranging from 20,613–30,376 baht, our study estimated this cost to be between 204,169 and 497,044 baht. However, it is important to note that a direct comparison between the two studies should be made with caution. It should be noted that the previous study [17] adopted the direct relationship between volume alcohol drinking and all-cause mortality in its estimates. However, all-cause mortality is a problematic endpoint, as causal associations of risk factors cannot be established, and consequently the results are biased [47,48]. In addition, it should be noted that non-chronic condition/disease including road injuries do not depend only on the volume of drinking but also pattern of drinking [49]. Unlike the previous study, our study considered the cause-specific risk of mortality from six main alcohol-attributable diseases/conditions in its analysis. When estimating cost associated with road injuries, which is non-chronic condition, we considered both volume and pattern of drinking.

In this study, the cost of alcohol consumption is significantly higher in males than in females for several reasons. Firstly, the average daily alcohol consumption in Thai males is roughly three times greater than in females (males consuming 30.1 g/day compared to 8.0 g/day in females [19]). Secondly, males have a higher risk of developing several alcohol-related diseases, especially hypertension, which is the leading factor contributing to the total costs of alcohol consumption in this analysis, even when drinking the same amount as females [24]. Additionally, the incidence rates of the six alcohol-related diseases were higher in males, even among never drinkers, likely due to greater engagement in risk behaviors. For instance, smoking prevalence in Thai males is 34.7%, compared to just 1.3% in females, while the drink-driving rate is also significantly higher among males (34.6% vs. 19.7% in females) [12]. Collectively, these factors contribute to a higher disease burden and earlier mortality in males, ultimately leading to increased lifetime costs of alcohol consumption and a distinct cost distribution compared to females.

With the current drinking patterns in Thailand (i.e., age at starting drinking was 20 for males and 23 years old for females, the average age at quitting was 55 for males and 49 years old for females, and that 24.59% of male drinkers and 21.18% of female drinkers were considered to be lifetime drinkers) [12], costs incurred from each individual drinker in Thailand was estimated at 117,895 baht from the healthcare-payer perspective and 498,196 baht from the societal perspective. These findings underscored the importance of the enforcement of existing alcohol policies as well as the introduction of innovative strategies to prevent individuals between the ages of 20 and 23 years old to become new drinkers. Given that 51.32% of male and 29.25% of female drinkers were considered underage (< 20 years old) at drinking onset [12], the costs associated with underage drinking are also warranted for further study. Meanwhile, alcohol preventive measures should also be targeted to this young population as it is expected that costs incurred from underaged drinkers would be even higher than this estimate.

Due to the dynamic nature of alcohol consumption patterns, reassessment on the lifetime cost of alcohol consumption to reflect the current economic impacts of drinking is also warranted, in order to support public health policy decision making. Nevertheless, drinking pattern in terms of age at start drinking during year 2017 [50] and 2021 [12] has not changed significantly. Additionally, when compared to the 2014 survey [51], the current drinking levels for both genders [19] remained within the “light alcohol drinking” category (female: > 0– < 20 g/day; male: > 0– < 40 g/day).

Similar to the previous study [17], the results from our scenario analysis indicated that the sooner drinkers quit drinking, the longer they live, and the greater the costs saved. In the male drinker, the costs saved from quitting drinking ranged from 529,288 baht when quitting occurred at the aged of 35–67,281 baht when quitting occurred at the age of 65. Similarly, the costs saved if a female drinker quit ranged from 232,865 baht when she quit at age 35–56,973 baht if she quit at age 65. Based on our findings, the interventions to reduce alcohol consumption should be aimed at the younger drinkers. While drinkers may not experience substantial benefits from alcohol abstinence in terms of life expectancy if they quit at older ages, advocating for alcohol abstinence, however, remains crucial for promoting better physical and mental health. As shown in our results, the direct medical cost was the largest component of the total costs saved from those drinkers who quit later on in life.

For lifetime drinkers, AUDs was the top leading cause of the cost associated with alcohol consumption (40.86% and 21.04% of the total excess cost for male and female lifetime drinkers, respectively). In contrast, hypertension had a significant impact on costs among non-lifetime drinkers (39.49% and 15.05% of the total excess cost for male and female non-lifetime drinkers, respectively). According to the Thai National Mental Health Survey 2013 [52], the prevalence of AUDs among those aged 18 and above was 5.3 percent, which accounted for approximately 2.7 million individuals in the country. For hypertension, it was estimated that the number of people in Thailand with hypertension in 2021 was approximately 14 million [19]. Of these, only around 50% were reported to be receiving treatment. These findings and statistics underscore the significant economic impacts of alcohol drinking on AUDs and hypertension and highlights the need for the targeted prevention, early intervention, and treatment efforts to reduce the incidence and prevalence of drinking to reduce the burden of AUDs and hypertension in the country. In addition, enhancing access to screening and treatment for AUDs and hypertension should be prioritized.

As indicated from our findings, cost saved from premature mortality accounted for the significant part for the total cost saved from quitting, accounting for 58.02% in male drinkers and 71.45% in female drinkers. Additionally, road injuries emerged as a major contributor to total costs in this study and a leading cause of premature deaths among young adults [53]. Given this impact, reducing alcohol-related road injuries should be a public health priority, as it could yield substantial economic benefits. Stricter enforcement of drink-driving laws, increased checkpoints, and targeted public campaigns should be implemented [54].

To address harmful alcohol use at the national level, the World Health Organization (WHO) has identified the five most cost-effective policy interventions. These include restricting access to alcohol through retail limitations, increasing alcohol taxes and prices, implementing bans on alcohol advertising, enforcing drink-driving laws, and providing brief interventions for hazardous drinking [55,56]. Recently, Thailand has implemented all these interventions, reflecting the country’s commitment to reducing the harmful effects of alcohol consumption through evidence-based strategies [57]. Nevertheless, a slightly increase in alcohol consumption was observed [13,58]. This could be due to the fact that the effectiveness of these alcohol policies is compromised by weak enforcement. The challenges in implementing and enforcing policies included a lack of coordination between government agencies, inadequate resource allocation, the absence of local community involvement, particularly in regulating the number of alcohol outlets, weak penalties, and low credibility of the law. Furthermore, while taxing alcoholic beverages was recognized as one of the most cost-effective policy options [59], tax rates in Thailand are not aligned with the current economic conditions [60,61]. To reduce the economic burden attributable to alcohol drinking in Thailand, there is a crucial need for robust law enforcement strategies [60].

Additionally, epidemiological evidence indicates that seriously practicing Buddhism is a protective factor for alcohol drinking among Thai high-school students [62] and men [63], primarily due to the religion’s teachings, which advocate abstinence from intoxicants as one of the Five Precepts. A 23-year time series analysis found that the Buddhist Lent Dry campaign, promoting temporary abstinence during the three-month Buddhist Lent Period (BLP), significantly reduced alcohol consumption at the population level [64], while a community-level study demonstrated that incorporating community activities during BLP further enhanced its effect on long-term abstinence [65]. Thus, integrating religious practices and community involvement into awareness campaigns and initiatives should be a key component of alcohol control strategies, particularly those aimed at young people.

To the best of our knowledge, our study represents the first study that comprehensively examines the lifetime cost of alcohol consumption, the costs that could be saved from quitting drinking at specific ages, and the costs incurred by the society for each individual drinker who drinks at the current level of drinking patterns. Our analysis also covered both direct and indirect costs. The annual transitional probability among lifetime abstainers as well as the direct medical costs and direct non-medical costs associated with each alcohol-related disease/condition were based on the Thai context.

Nevertheless, several limitations of the study should be addressed. Firstly, our analysis only provided an estimate of the costs associated with drinkers who start drinking at specific ages and maintain the same quantity over their entire lifespan or for specific time intervals. Therefore, the findings may not apply to populations with different drinking behaviors. However, while alcohol consumption in Thailand varied by region and age [19,51]), it is important to note that both male and female drinkers in Thailand, regardless of region and age, were typically classified as “light alcohol drinkers” (females < 20 g/day; males < 40 g/day). Therefore, our findings can generally be applied to drinkers across all regions of the country. Secondly, cost of underage drinking was not considered in our analysis. Given that some individuals begin drinking before the age of 20, further research is needed to assess the costs associated with underage drinking. Thirdly, the model’s simplifications may lead to underestimation of costs, life year losses, and uncertainty in results. As only six alcohol-related conditions were considered in the model, the costs in this study were likely to be underestimated. Nevertheless, these six alcohol-related diseases/conditions included in the model represented 70.16% of the total alcohol-related disease burden in Thai males and 76.13% in Thai females [11]. Furthermore, only five cost components were considered. Further exploration of other indirect costs, such as long-term caregiving or productivity loss outside of absenteeism, could provide a more comprehensive understanding of the economic burden. In addition, this study included some alcohol-related parameters collected during the COVID-19 pandemic, which may not reflect typical alcohol consumption patterns. It was also important to note that the former drinkers in the study were immediately presumed to possess the same risk as the lifetime abstainers. This assumption was made for several reasons, including data availability and the effect of “sick quitting”, a situation where an individual stops drinking alcohol because they experience negative health consequences due to drinking, rather than as a result of voluntary quitting. Additionally, it should be noted that the risk reduction after alcohol cessation depends on the duration of abstinence and the specific disease [66]. For instance, the risk of liver cancer gradually decreases after cessation [66] while risk of alcohol-related injuries declines immediately after cessation, as the cognitive and motor impairments caused by intoxication are eliminated [2]. Nevertheless, since the way disease risk changes over time for former drinkers remains a relatively underexplored area with substantial uncertainty [66], former drinkers in the study were assumed to have the same risk as lifetime abstainers right after they stopped drinking. While we acknowledged that this assumption likely resulted in an overestimation of the costs saved from quitting, we believed that it has no or minimal effect on costs associated from road injuries and hypertension, which were main cost drivers in the estimates. Importantly, further research is warranted to estimate the effect of drinking among former drinkers. Another limitation is that many assumptions were unavoidably required in our analysis to balance between the intricate dynamics of disease progression, data availability, and modeling techniques. While these limitations were comprehensively discussed in the study protocol [18], it should be noted that a number of sensitivity analyses conducted in this study provided valuable insights into the robustness of the results and the potential impact of these parameter variations, guiding future research and policy considerations. Moreover, face validity and external validation were also performed to ensure that the results from the model were reasonable. Lastly, while our study provides evidence on the economic benefits of preventing alcohol initiation and encouraging alcohol cessation, which could support investments in several cost-effective alcohol policies (e.g., raising taxes, banning advertising, and enforcing drink-driving laws) [67–69], further cost-effectiveness studies are necessary to justify public health investments in various alcohol control interventions.

## Conclusion

The economic burden incurred by the society for each individual drinker is substantial. To lessen the economic burden of alcohol consumption in the country, the government and all stakeholders should focus on the development of innovative strategies such as regulating outlet density as well as the implementation of well-formulated law enforcement protocols on various cost-effectiveness measures to prevent individuals from becoming drinkers and to support individual drinkers to quit drinking at the earliest age possible. These included restricting alcohol marketing, rising alcohol tax, and increase access to counseling and treatment.

## Supporting information

S1 File Supplementary. (PDF)
