# Correction: What is the lifetime cost of alcohol consumption? An estimation of economic burden in Thailand

**DOI:** 10.1371/journal.pone.0331594

**Published:** 2025-09-04

**Authors:** The PLOS One Staff

The Funding statement for this article is incorrect. The correct Funding statement is as follows: This study is part of a PhD research project in the Social, Economic, and Administrative Pharmacy graduate program of C.L., whose scholarship was provided by the RGJ-Ph.D. Program under the National Research Council of Thailand, Ministry of Higher Education, Science, Research, and Innovation (Grant No. PHD/0061/2561). The grantor of the scholarship had no role in the study design, data collection, analysis, or interpretation, manuscript writing, or the decision to publish the results.

The publisher apologizes for the error.
