# Supplementary material for: What is the lifetime cost of alcohol consumption? an estimation of economic burden in Thailand
Source: PLoS One. 2025 May 16;20(5):e0322944. doi: 10.1371/journal.pone.0322944 (PMC12083796; doi:10.1371/journal.pone.0322944)
Supplement: S1 File — (PDF) [file pone.0322944.s001.pdf]

Supplementary

What is the Lifetime Cost of Alcohol Consumption? An Estimation of Economic Burden in Thailand

Chaisiri Luangsinsiri[1,2], Montarat Thavorncharoensap[3,4]*, Usa Chaikledkaew[3,4], Oraluck Pattanaprateep[5], Naiyana Praditsitthikorn[6], Bundit Sornpaisarn[2,7], Jürgen Rehm[2,7]

[1] Doctor of Philosophy Program in Social, Economic, and Administrative Pharmacy, Department of Pharmacy, Faculty of Pharmacy, Mahidol University, Bangkok, Thailand

[2] Institute for Mental Health Policy Research, Centre for Addiction and Mental Health, Toronto, Canada

[3] Mahidol University Health Technology Assessment (MUHTA) International Graduate Program, Mahidol University, Bangkok, Thailand

[4] Social and Administrative Pharmacy Excellence Research (SAPER) unit, Department of Pharmacy, Faculty of Pharmacy, Mahidol University, Bangkok, Thailand

[5] Department of Clinical Epidemiology and Biostatistics, Faculty of Medicine Ramathibodi Hospital, Mahidol University, Thailand

[6] Department of Disease Control, Ministry of Public Health, Nonthaburi, Thailand

[7] Dalla Lana School of Public Health, University of Toronto, Toronto, Canada

*Corresponding Author:
Email: montarat.tha@mahidol.ac.th (MT)

S1 Table. Model input parameters.

Parameter	Value*		Reference
	Male	Female	
Proportion of non-fatal injury by severity			
Minor injury	0.77130	0.77130	[1]
Severe injury	0.21920	0.21920	[1]
Disability	0.00950	0.00950	[1]
Annual cost of alcohol-related diseases (baht)			
Hypertension	9,963	9,375	NHSO database
Hemorrhagic stroke	58,582	57,944	[2]
Liver cirrhosis	76,409	76,409	[3]
Liver cancer	86,210	78,125	NHSO database
AUDs	16,734	14,322	NHSO database
Road injury with disability (1st year)	9,337	9,337	[1]
Road injury with disability (Long term)	311,142	311,142	[1]
Severe road injury	25,156	25,156	[1]
Minor road injury	778	778	[1]
Fatal road injury	16,626	16,626	[1]
Non-medication of outpatients per visit	145	145	[4]
Non-medication of inpatients per day	229	229	[4]
Property damage due to road traffic accidents per case	757	757	[5]
Annual hospital-related absent day			
1. Number of OPD visit per person per year			
Hypertension	3.08	3.26	NHSO database
Hemorrhagic stroke	2.87	2.87	NHSO database
Liver cirrhosis	2.42	2.56	NHSO database
Liver cancer	3.75	3.61	NHSO database
AUDs	2.38	1.91	NHSO database
Road injury	1.00	1.00	Assumption
2. Length of hospital stay per person per year (days)			
Hypertension	2.47	2.33	NHSO database
Hemorrhagic stroke	6.66	6.54	NHSO database
Liver cirrhosis	5.28	5.63	NHSO database
Liver cancer	8.02	8.44	NHSO database
AUDs	5.32	4.43	NHSO database
Road injury	1.31	0.75	NHSO database
Proportion of alcohol drinker by drinking status			
Lifetime abstainer among gen pop	0.28148	0.78585	[6]
Ever drinker among gen pop	0.71852	0.21415	[6]
Lifetime drinker among ever drinkers	0.24586	0.21177	[6]
Non-lifetime drinker among ever drinkers	0.75414	0.78823	[6]
Other parameters			
GNI per capita in 2022 (baht)	248,677	248,677	[7]
GNI growth rate (20-year average, %)	2.73	2.73	[8]
Discount rate (%)	3.00	3.00	[9]

Gen pop General population; *GNI* Gross National Income; *NHSO* National Health Security Office, Thailand.
*Value varied by age.

S2 Table. The percentage of costs of alcohol consumption for lifetime. abstainers, lifetime drinkers, and non-lifetime drinkers, and the percentage of the excess costs of alcohol consumption, by disease/condition in baht (2022).

Disease	LC	LCA	HT	HS	RI	AUDs	Other diseases*
**The total costs of alcohol consumption among lifetime abstainers**							
**Male**	1.98%	0.64%	75.80%	3.14%	0.05%	0.00%	18.39%
**Female**	3.88%	1.29%	71.10%	4.08%	0.25%	0.00%	19.41%
**The total costs of alcohol consumption among lifetime drinkers**							
**Male**	2.99%	0.77%	63.56%	4.14%	5.04%	14.32%	9.18%
**Female**	5.42%	1.84%	61.80%	6.26%	6.19%	3.58%	14.91%
**The total costs of alcohol consumption among non-lifetime drinkers**							
**Male**	2.29%	0.69%	67.26%	3.81%	5.11%	10.37%	10.48%
**Female**	4.00%	1.39%	66.99%	4.81%	3.56%	1.64%	17.62%
**The excess costs of alcohol consumption for lifetime/non-lifetime drinkers compared to lifetime abstainers****							
**Male**							
**LD**	4.68%	0.94%	33.55%	5.68%	14.29%	40.86%	-
**nLD**	2.82%	0.73%	39.49%	5.09%	17.03%	34.84%	-
**Female**							
**LD**	12.61%	4.41%	10.26%	16.57%	35.11%	21.04%	-
**nLD**	5.27%	2.57%	15.05%	13.34%	42.75%	21.02%	-

LD Lifetime drinker; *nLD* Non-lifetime drinker; *HT* hypertension; *HS* hemorrhagic stroke; *LC* liver cirrhosis; *LCA* liver cancer; *AUDs* alcohol use disorders; *RI* road injury.

*Only costs of premature mortality were included in the analysis.

**The percentages were calculated using the total costs, from which the costs of premature mortality caused by the other diseases in the model have not been subtracted, as the dominators.

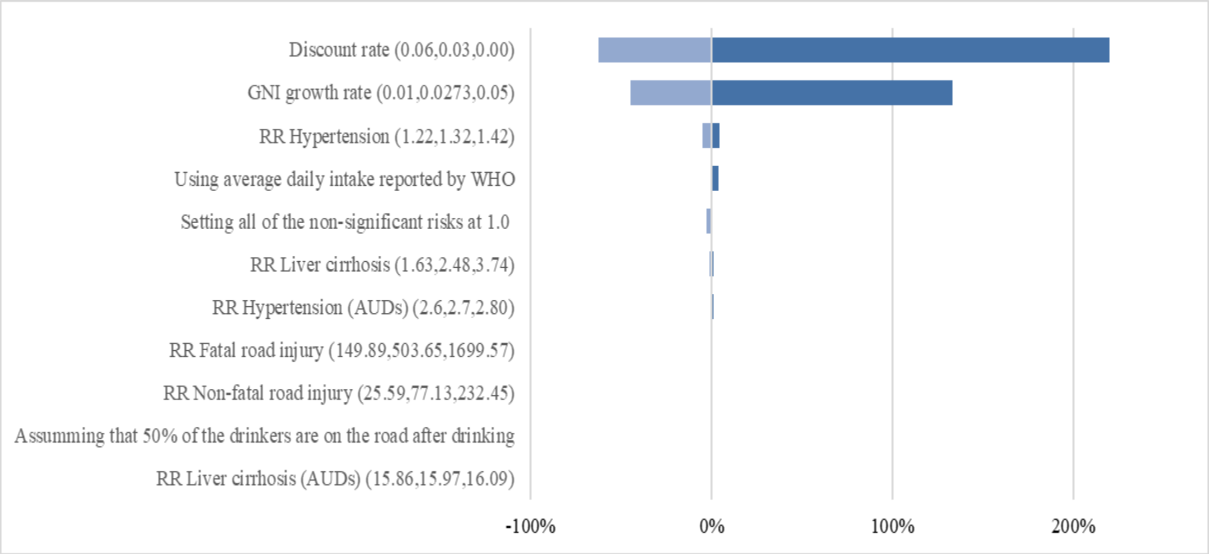

S1 Fig. The tornado diagram for lifetime male drinkers.

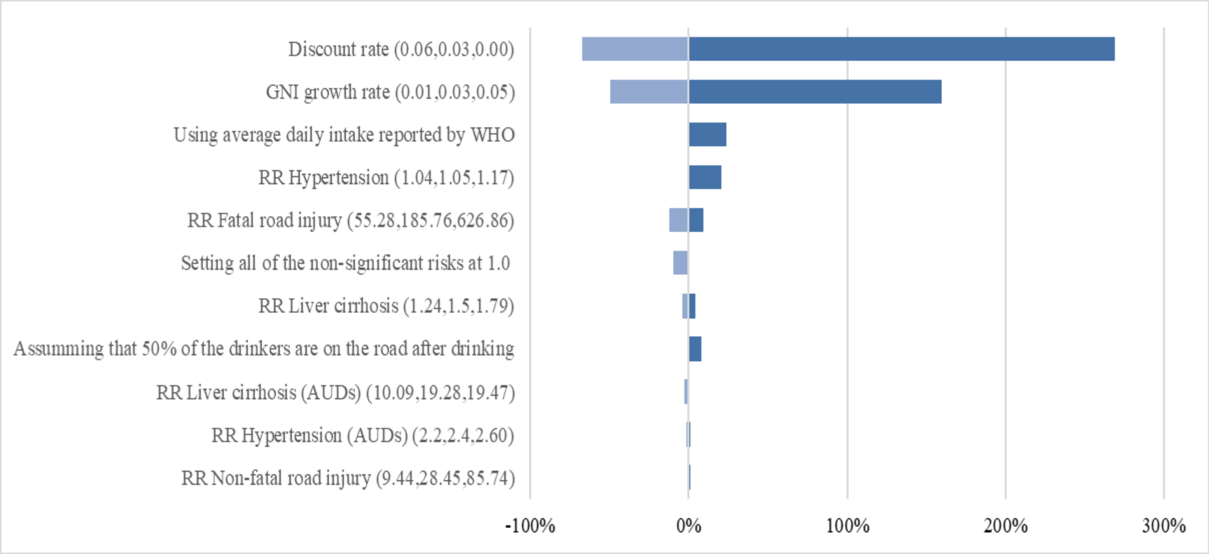

S2 Fig. The tornado diagram for lifetime female drinkers.

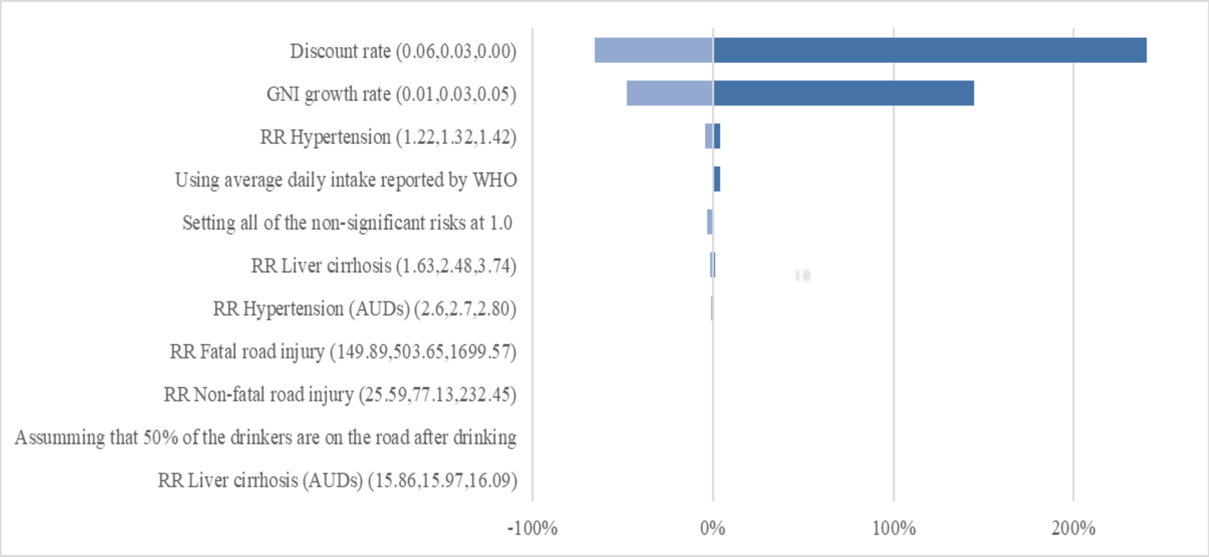

S3 Fig. The tornado diagram for male individuals who quit drinking at the age of 35.

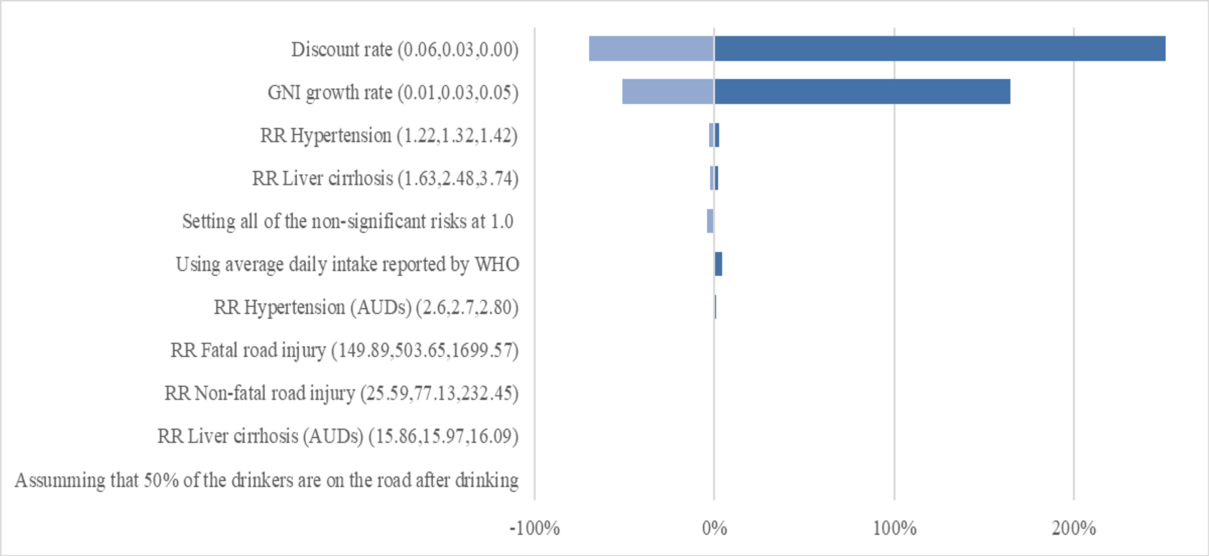

S4 Fig. The tornado diagram for male individuals who quit drinking at the age of 45.

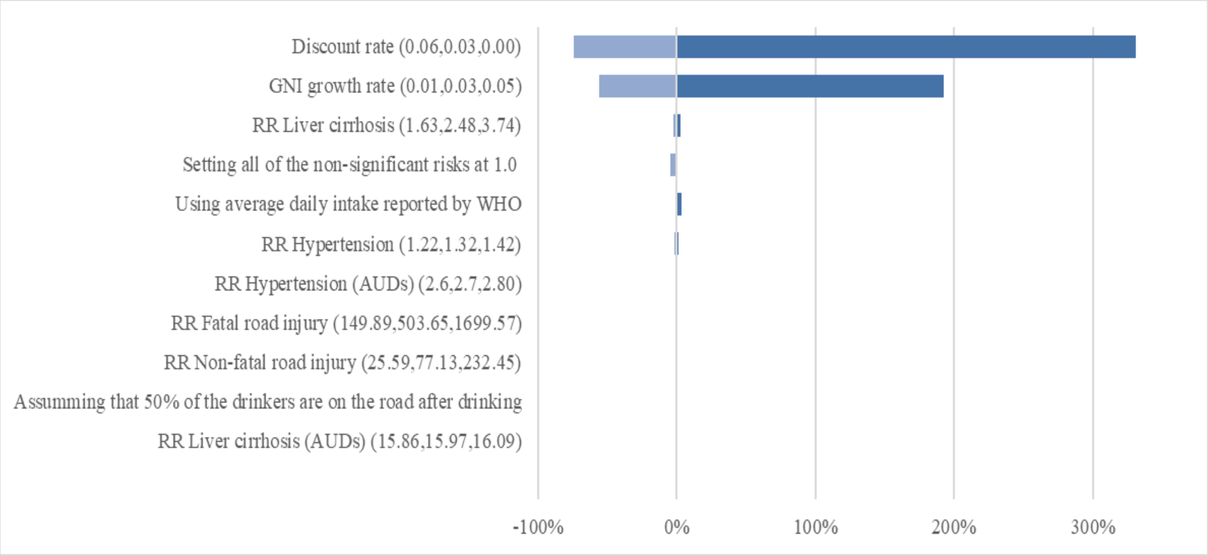

S5 Fig. The tornado diagram for male individuals who quit drinking at the age of 55.

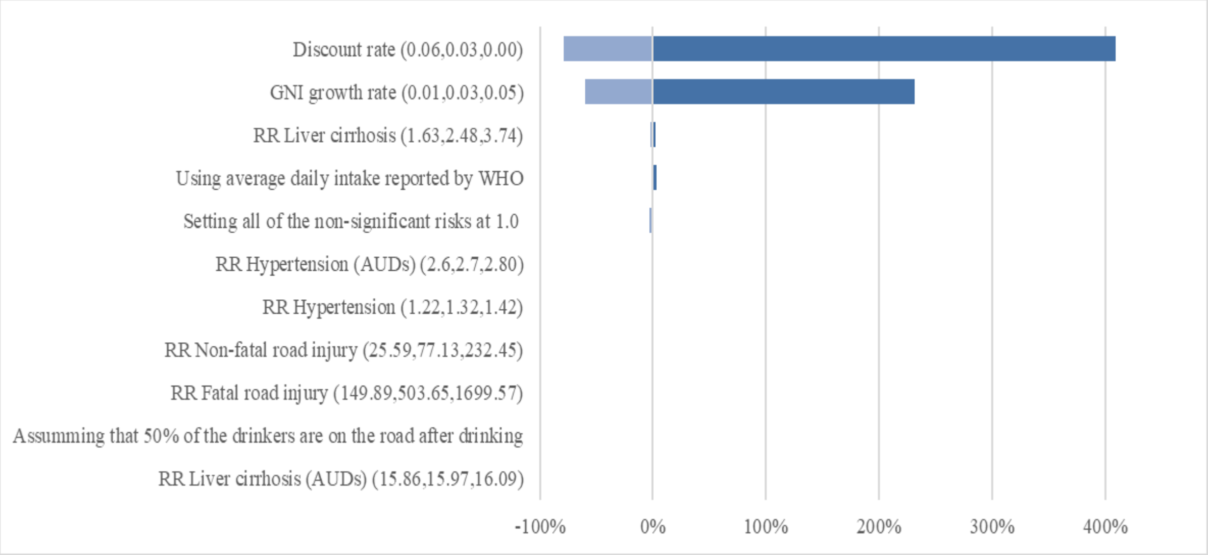

S6 Fig. The tornado diagram for male individuals who quit drinking at the age of 65.

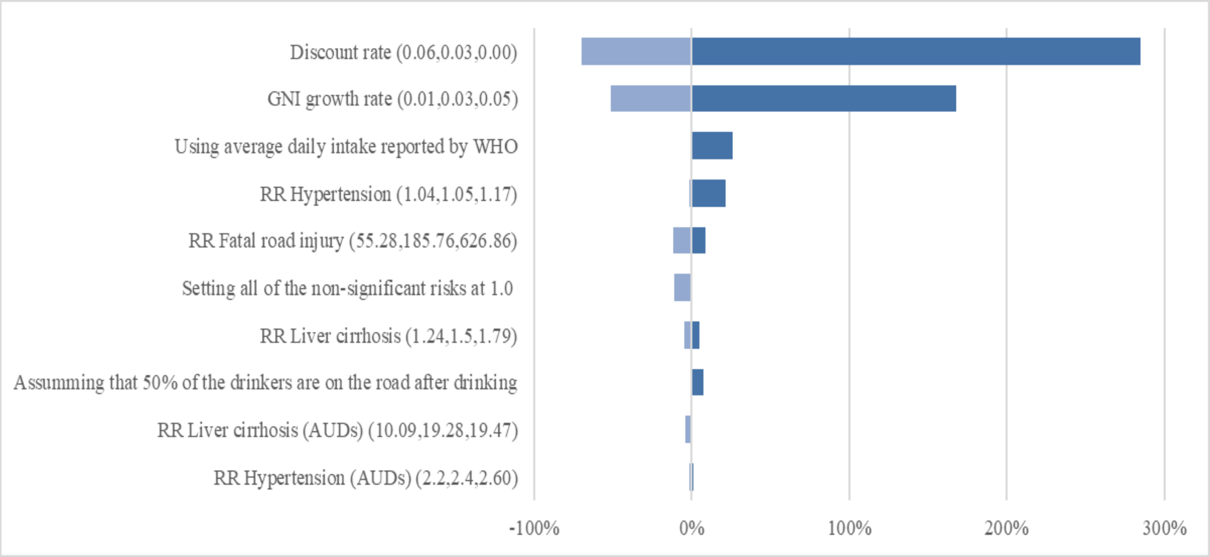

S7 Fig. The tornado diagram for female individuals who quit drinking at the age of 35.

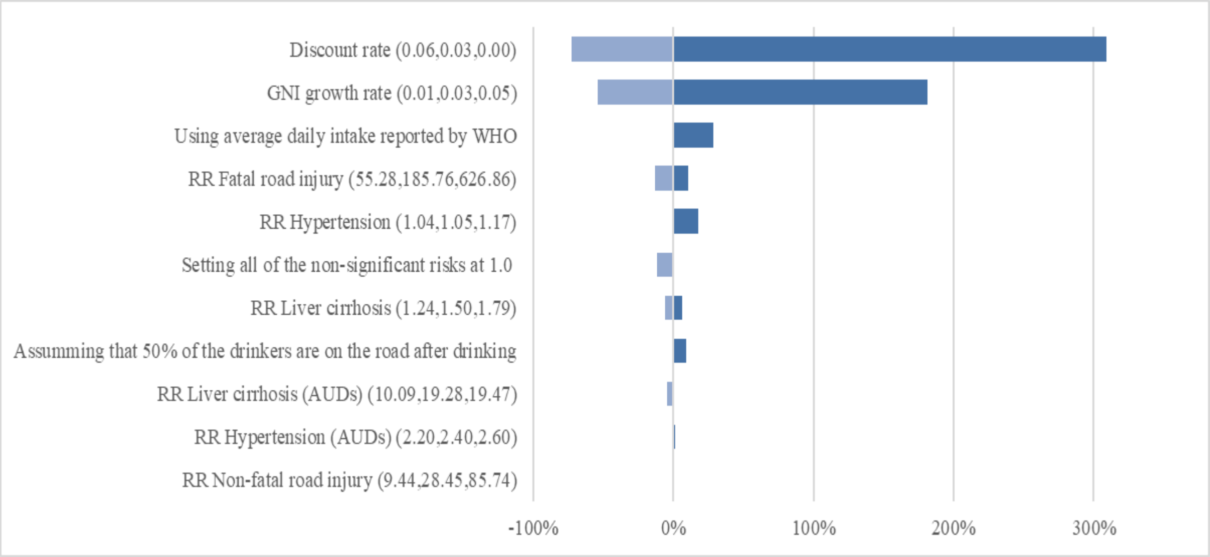

S8 Fig. The tornado diagram for female individuals who quit drinking at the age of 45.

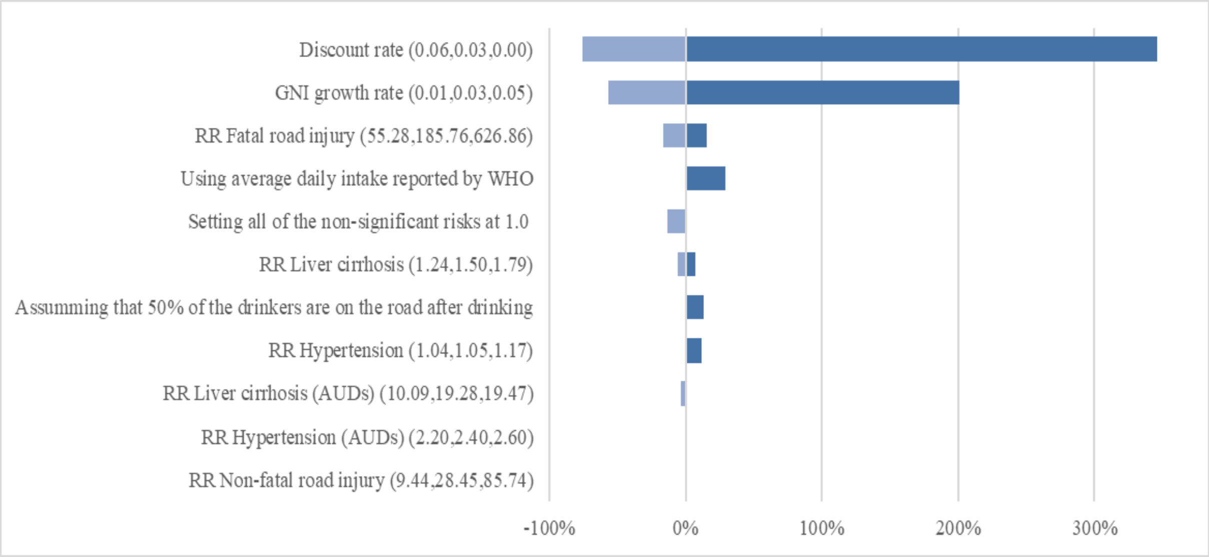

S9 Fig. The tornado diagram for female individuals who quit drinking at the age of 55.

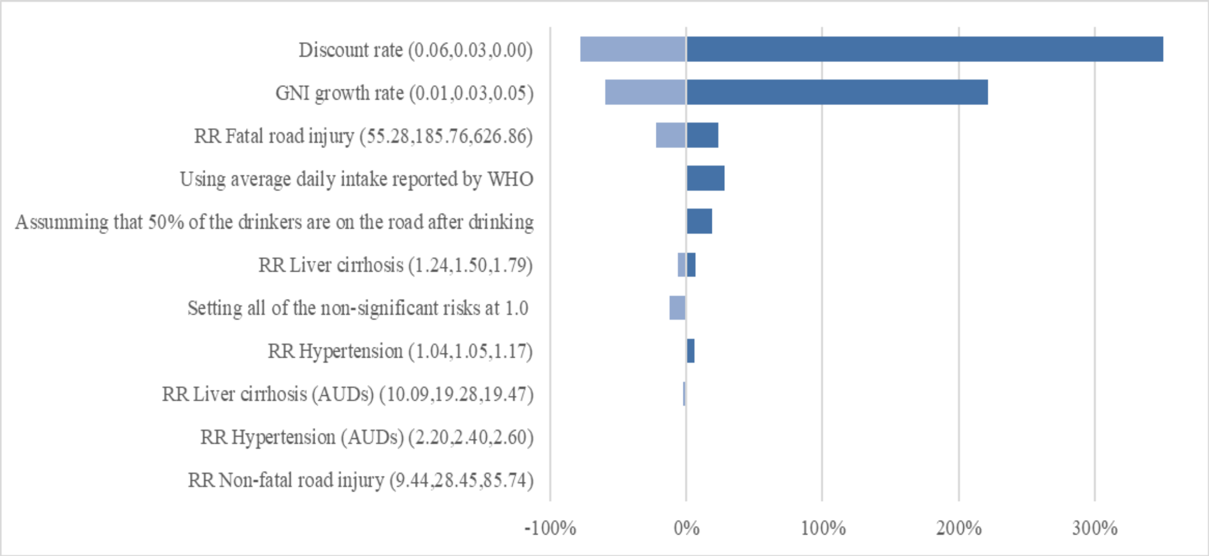

S10 Fig. The tornado diagram for female individuals who quit drinking at the age of 65.

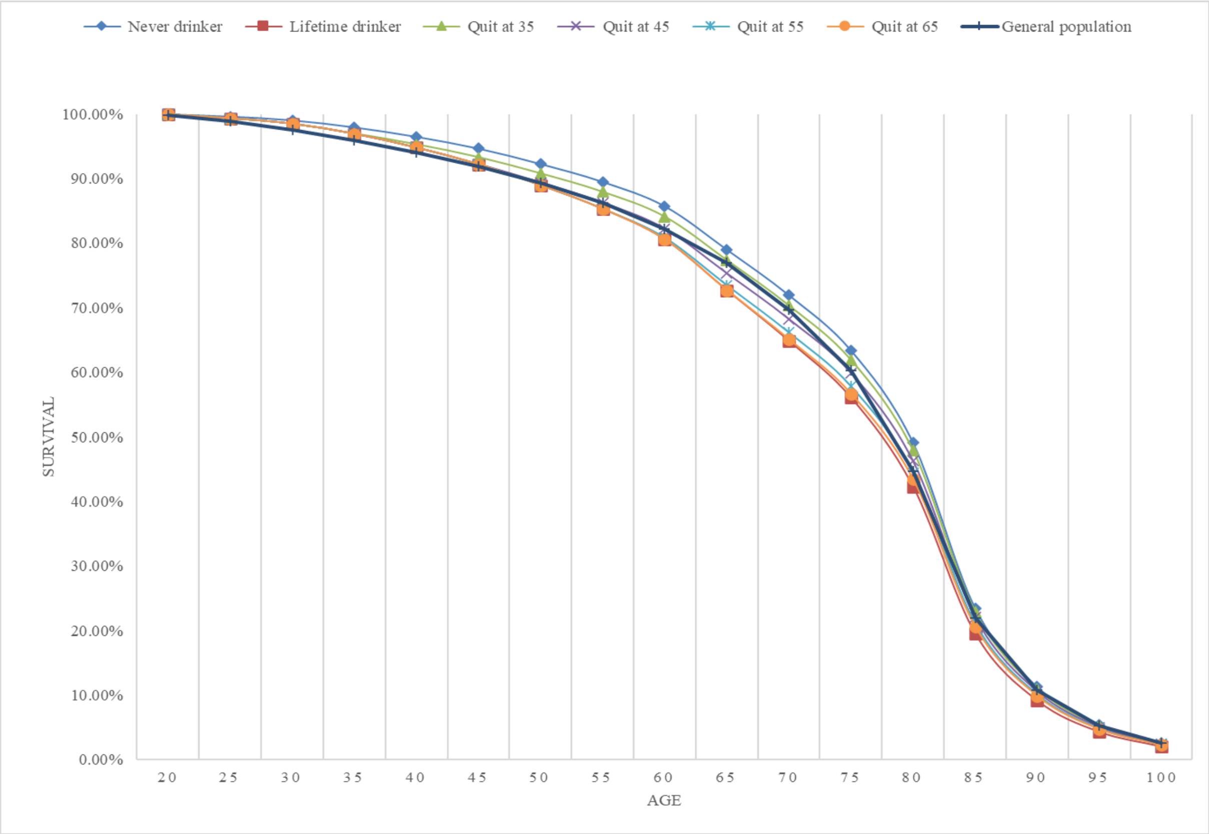

S11 Fig. Survival curve in male individuals by drinking pattern.

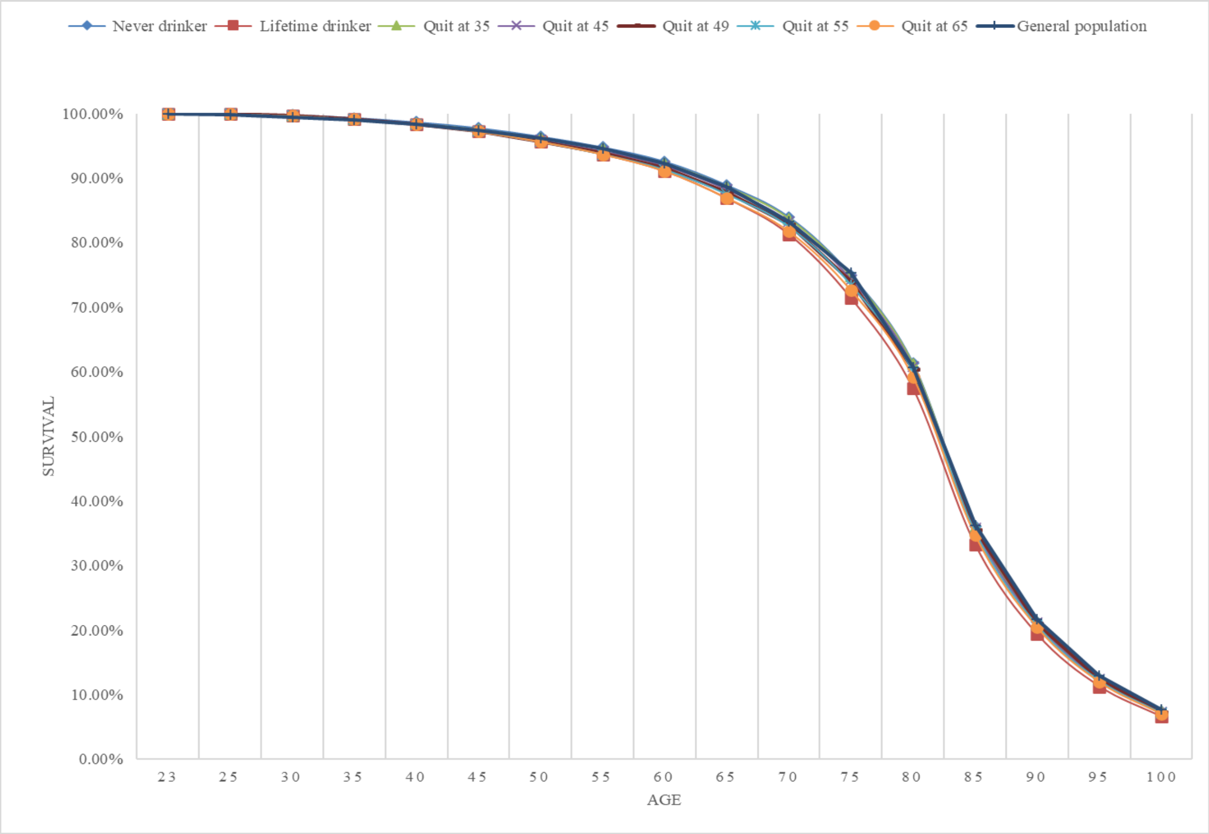

S12 Fig. Survival curve in female individuals by drinking pattern.

References

1. Faculty of Engineering, Prince of Songkla University. The study of traffic accident costs in Thailand. Songkla: Department of Highways, Ministry of Transportation; 2007.
2. Kumluang S, Wu O, Langhorne P, Geue C. Stroke resource utilisation and all-cause mortality in Thailand 2017-2020: A retrospective, cross-sectional study. BMJ Open. 2023;13(6):e072259. doi: https://doi.org/10.1136/bmjopen-2023-072259. PubMed PMID: 37280024; PubMed Central PMCID: PMCPMC10255610.
3. Poovorawan K, Treeprasertsuk S, Thepsuthammarat K, Wilairatana P, Kitsahawong B, Phaosawasdi K. The burden of cirrhosis and impact of universal coverage public health care system in Thailand: Nationwide study. Ann Hepatol. 2015;14(6):862-8. doi: https://doi.org/10.5604/16652681.1171773. PubMed PMID: 26436358.
4. Riewpaiboon A. Standard cost lists for health economic evaluation in Thailand. J Med Assoc Thai. 2014;97 Suppl 5:S127-34. PubMed PMID: 24964710.
5. The Royal Thai Police. Statistics of road traffic accident casualty and property damaged 2022. Available from: https://ittdashboard.nso.go.th/preview.php?id_project=64.
6. National Statistical Office. The 2021 Heath Behavior of Population Survey. Bangkok: Statistical Forecasting Division; 2021.
7. Office of the national economic and social development council. Report on economic conditions in Thailand (Q1/2023) 2023 [cited 2023 20 May]. Available from: https://www.nesdc.go.th/more_news.php?cid=871&filename=index.
8. The World Bank. GDP per capita growth (annual %)-Thailand 2022 [cited 2023 20 May]. Available from: https://data.worldbank.org/indicator/NY.GDP.PCAP.KD.ZG?locations=TH.
9. Guideline Development Working Group. Guideline for health technology assessment in Thailand updated edition: 2019. Bangkok: Health Systems Research Institute; 2021.
